# High frequency of myeloid-derived suppressor cells in sepsis patients, with the granulocytic subtype dominating in Gram-positive cases

**DOI:** 10.1186/cc14006

**Published:** 2014-12-03

**Authors:** H Janols, C Bergenfelz, R Allaoui, A-M Larsson, L Rydén, S Björnsson, S Janciauskiene, M Wullt, A Bredberg, K Leandersson

**Affiliations:** 1 Department of Infectious Diseases, Skane University Hospital, Lund University, Malmo, Sweden; 2 Center for Molecular Pathology, Skane University Hospital, Lund University, Malmo, Sweden; 3 Department of Laboratory Medicine, TCR, MV, Lund University, Lund, Sweden; 4 Department of Surgery, Lund University Hospital, SUS, Lund, Sweden; 5 Cytometry Laboratory and Department of Laboratory Medicine, Skane University Hospital, Lund University, Malmo, Sweden; 6 Department of Pulmonology, Hannover Medical School, Hannover, Germany; 7 Department of Medical Microbiology, Skane University Hospital, Lund University, Malmo, Sweden

## Introduction

Myeloid-derived suppressor cells (MDSCs) constitute a heterogeneous population of immature myeloid cells that potently suppress immune responses. They were originally identified in cancer patients and have since been reported to occur also in chronic inflammation, autoimmunity and even bacterial infections. Human MDSCs are commonly divided into monocytic (Mo-MDSCs) and granulocytic (PMN-MDSCs) subtypes. To what extent the *bona fide *cancer MDSCs are representative of the proposed MDSCs found in other diseases is not well known. PMN-MDSCs have previously been found to be enriched among low-density granulocytes (LDGs) in density gradient centrifuged blood.

## Methods

In this study we analyzed potential MDSCs in sepsis patients with different causative microorganisms, using total peripheral blood as compared to density gradient centrifuged blood.

## Results

**Figure 1:**
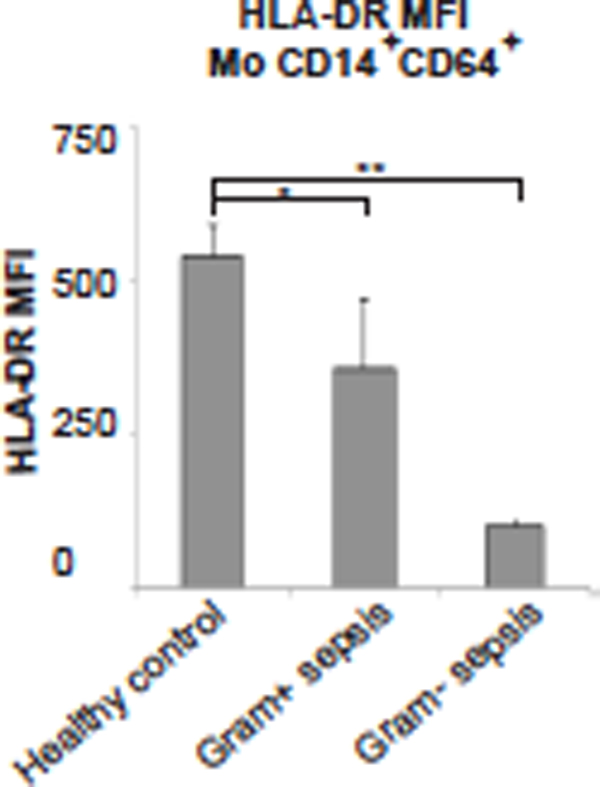


**Figure 2:**
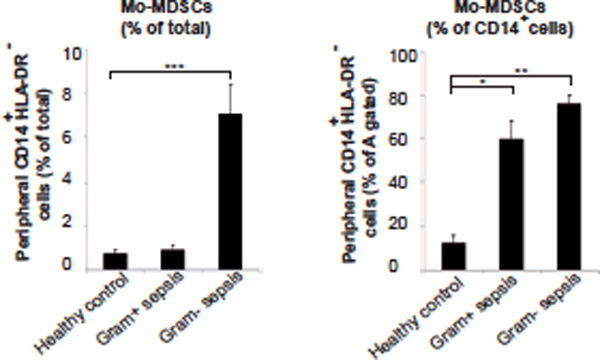


**Figure 3:**
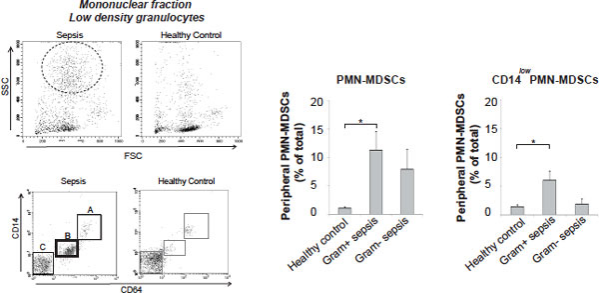


**Figure 4:**
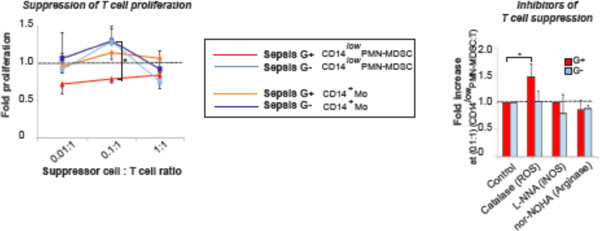


**Figure 5:**
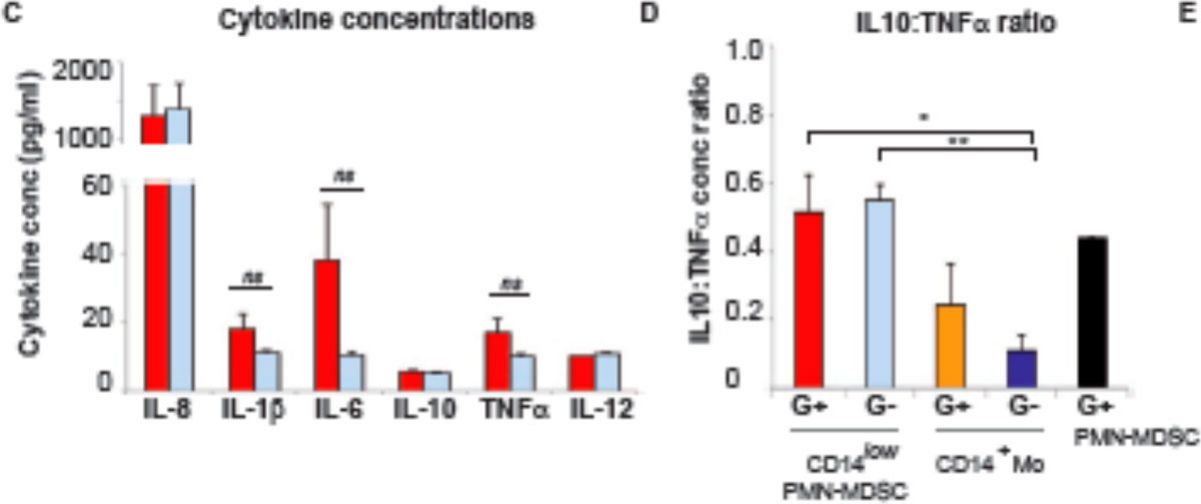


We found a high frequency of typical CD14^+^HLADR^low ^Mo-MDSCs in all sepsis patients, whereas the typical PMN-MDSCs as well as a prominent CD14^low ^PMN-MDSC-like population appeared preferentially in Gram-positive cases (Figures 1 to 3). The CD14^low ^PMN-MDSC variant was demonstrated to suppress T-cell proliferation *in vitro *via a ROS-dependent mechanism, to display an increased IL-10:TNFα ratio, and to present with signs of immaturity: blast morphology and low cytokine levels (Figures 4 and 5).

## Conclusion

We conclude that a spectrum of cells with MDSC features are enriched in sepsis, and that microbial origin of sepsis contributes to the substantial interindividual patient variation in MDSC pattern.

